# Patients with precursor disease exhibit similar psychological distress and mental HRQOL as patients with active myeloma

**DOI:** 10.1038/s41408-019-0172-1

**Published:** 2019-01-21

**Authors:** Imad Maatouk, Susanne He, Manuela Hummel, Stefan Hemmer, Michaela Hillengass, Hartmut Goldschmidt, Mechthild Hartmann, Wolfgang Herzog, Jens Hillengass

**Affiliations:** 1 Department of General Internal Medicine and Psychosomatics, Medical University Hospital, Heidelberg, Germany; 2 National Center for Tumor Diseases, Heidelberg, Germany; 3 Department of Hematology, Oncology, and Rheumatology, Medical University Hospital, Heidelberg, Germany; 4 Department of Biostatistics, German Cancer Research Center, Heidelberg, Germany; 5 Department of Orthopedics and Traumatology, University Hospital, Heidelberg, Germany; 6 Roswell Park Comprehensive Cancer Center, Clinical Research Service, Buffalo, NY USA; 7 National Center for Tumor Diseases, Section of Multiple Myeloma, Heidelberg, Germany; 8 Department of Medicine, Roswell Park Comprehensive Cancer Center, Buffalo, NY USA

Multiple myeloma (MM) is largely a disease of the elderly with a median age at first diagnosis of 69 years. MM accounts for 10% of hematologic malignancies, with an incidence of 5–8/100,000 individuals in the US^1^. Several studies have investigated the impact of MM on health-related quality of life (HRQOL), psychological distress (DT), and mental comorbidity^2^ due to a variety of disease- and treatment-related events leading to impairment in terms of HRQOL and increased DT^3^.

However, MM is consistently preceded by a monoclonal gammopathy of undetermined significance (MGUS) and smoldering (asymptomatic) MM (SMM), which are premalignant stages of the disease with the presence of monoclonal plasma cells and/or monoclonal protein, but lacking so-called myeloma-defining events^4^.

It is estimated that ~5% of the elderly population (aged ≥70 years) are diagnosed with MGUS. In individuals aged ≥85 years the prevalence of MGUS in men increases to 9%. Depending on clinical risk factors, MGUS and SMM require regular consultations, as the annual risk of progression is 1 and 10% for MGUS and SMM, respectively^5^. Clinicians and patients report that this period of watchful “waiting” characterized by repeated follow-up visits has a high psychosocial impact on patients’ quality of life. Pilot data suggest similar or even higher levels of DT in patients with non-malignant hematologic entities^6,7^. Here, we aimed to compare HRQOL, DT, anxiety, and depression between patients with MGUS/SMM and patients with MM pre- and post-treatment.

All patients who made the first appointment at our outpatient clinic between November 2014 and September 2017 received a letter prior to their appointment at the hospital. This letter included information on the survey and a psychosocial questionnaire package. Patients with a diagnosis of MM, SMM, or MGUS, and who also had sufficient skills in reading and writing German were eligible for study participation. Written, informed consent for analysis of patient data was obtained from all participants. Approval was obtained from the local ethics committee at the University Hospital of Heidelberg.

HRQOL was measured using the Short Form General Health Survey (SF-12).The items were weighted and totaled to provide both a physical component score (PCS) and a mental component score (MCS)^8^.

DT was measured using the National Comprehensive Cancer Network (NCCN) distress thermometer^9^.

The nine-item patient health questionnaire (PHQ-9) and the seven-item general anxiety disease-screening instrument (GAD-7) were used to measure depression and anxiety symptoms, respectively^10^.

Participants were classified into three groups based on the guidelines of the International Myeloma Working Group and according to time-point in the course of the disease as follows: new diagnosis of MM (pre-treatment), treated MM, and premalignant disease (MGUS or SMM)^4^.

Group comparisons (Kruskal–Wallis tests) for the respective outcomes and multivariate regression analyses were performed, with HRQOL, DT, anxiety, and depression scores as outcome measures.

Classification of a disease stage (Group 1: premalignant disease; reference, Group 2: new diagnosis of MM before treatment, and Group 3: treated MM) served as independent variable of interest in all multivariate regression analyses. All statistical analyses were performed using R for Windows (version 3.3.2).

Between November 2014 and September 2017, 552 of 1066 eligible patients (response rate: 51.8%) were enrolled in the study. The mean age was 62.1 years with a range of 30.7–87.5 years. The majority of patients were male (*n* = 324; 58.7%). Of the 552 patients, 190 (35.8%) had a new diagnosis of active MM, 193 patients (36.4%) had already been treated for MM, and 148 patients (27.9%) had a diagnosis of a precursor (MGUS or SMM) of MM. The mean values of the SF-12 sum scores in the whole cohort were 42.4 for MCS (range 11.5–68.9) and 39.1 for PCS (range 16.4–59.6), respectively. Psychological distress according to the DT level was above the threshold of ≥5 in 338 participants (63.9%). Moderate to severe anxiety symptoms according to the GAD-7 were prevalent in 12.4% of participants, while moderate to severe depressive symptoms according to the PHQ-9 were prevalent in 21% of participants. Characteristics of the entire sample are shown in Table 1.

**Table 1:** Patient characteristics

Variables	*M* (SD)	*n*	%
Age in years	62.1(10.5)	552	100
*Sex*			
Female		228	41.3
Male		324	58.7
*Stage of myeloma*			
Precusor disease (MGUS. SMM) (Group 1)		148	27.9
New diagnosis of MM (Group 2)		190	35.8
Treated MM (Group 3)		193	36.4
*Comorbidity*			
Charlson comorbidity index ≤2		404	74.1
Charlson comorbidity index >2		141	25.9
Distress thermometer	5.4 (2.8)	529	
Low (<5)		191	36.1
High (≥5)		338	63.9
*Health-related quality of life*			
Physical component score (PCS 12)	39.1 (11.0)	541	
Mental component score (MCS 12)	42.4 (11.6)	541	
Anxiety (GAD-7) score	4.5 (4.4)	532	
*Anxiety level*			
None (0–4)		323	60.8
Mild (4–9)		142	26.7
Medium (10–14)		40	7.5
Severe (15–21)		26	4.9
Depression (PHQ-9) score	5.9 (4.6)	542	
Depression level			
None (0–4)		247	45.6
Mild (4–9)		181	33.4
Medium (10–14)		84	15.5
Severe (15–27)		30	5.5

MM multiple myeloma, MGUS monoclonal gammopathy of undetermined significance, SMM smoldering myeloma, PCS physical component score, MCS mental component score, GAD-7 generalized anxiety disorder scale-7, PHQ-9 patient health questionnaire-9

Group comparison revealed that individuals with a precursor of MM reported higher physical HRQOL scores (PCS: mean 43.6) (*p* < 0.001) than patients with a newly diagnosed symptomatic stage of the disease (PCS: mean 39.1) and already treated patients (PCS: mean 35.7).

No statistically significant difference was found with regard to mental HRQOL in individuals with a precursor of MM (MCS: mean 43.1), newly diagnosed, (MCS: mean 42.9) and treated MM (MCS: mean 41.3) (*p* = 0.18). The outcome parameters of patients stratified by groups 1–3 with the respective *p*-values of group comparison tests are available as Supplementary Information (SI) on blood cancer journal's website.

The results were confirmed in multivariate analysis. The stage of the disease according to the above-mentioned classification was significantly related to PCS, but was not significantly associated with MCS, respectively. The results of all multivariate linear regression analyses are shown in Table 2.

**Table 2:** Multiple linear regression analyses with stage of disease as an independent variable and different outcome variables

Variable	HRQOL (PCS)		HRQOL (MCS)		Distress score		Anxiety score		Depression score	
	*B* [95%-CI]	*P*	*B* [95%-CI]	*P*	*B* [95%-CI]	*P*	*B* [95%-CI]	*P*	*B* [95%-CI]	*P*
Charlson index	−2.21 [−4.37; −0.06]	.04*	−3.79 [−6.16; −1.42]	.002*	0.48 [−0.10; 1.06]	.1	0.88 [−0.03; 1.79]	.06	1.54 [0.61; 2.47]	.001*
Age	−0.31 [−0.76; 0.14]	.17	0.57 [0.08; 1.07]	.02*	−0.14 [−0.26; −0.02]	.02*	−0.17 [−0.37; 0.02]	.07	−0.09 [−0.28; 0.10]	.37
Sex	−0.05 [−1.91; 1.81]	.96	3.53 [1.49; 5.57]	<.001*	−0.39 [−0.89; 0.11]	0.13	−0.57 [−1.36; 0.22]	.91	−0.70 [−1.50; 0.10]	.09
*Stage of myeloma*										
New diagnosis of MM	−2.63 [−5.09; −0.17]	.04*	1.39 [−1.31; 4.09]	.31	0.06 [−0.61; 0.72]	.87	0.28 [−0.77; 1.33]	0.6	0.01[−1.05; 1.07]	.98
Treated MM	−5.76 [−8.25; −3.27]	<.001*	−0.17 [−2.91; 2.56]	.9	−0.34 [−1.01; 0.34]	.33	0.06 [−1.00; 1.12]	.91	1.30 [0.22; 2.38]	.02*
Hb	0.91 [0.40; 1.41]	<.001*	0.41 [−0.14; 0.97]	.14	−0.21 [−0.35; −0.08]	.002*	−0.12 [−0.33; 0.09]	.27	−0.22 [−0.43; 0.00]	.05*

*MM* multiple myeloma, *HRQOL* health-related quality of life, *PCS* physical component score, *MCS* mental component score, *Hb* hemoglobin

Charlson-Index reference: ≤2; age reference: 5-year increase; sex reference: female; stage of myeloma reference: monoclonal gammopathy of undetermined significance/smoldering myeloma

When comparing psychological distress between patients with a precursor of MM (DT: mean 5.2), newly diagnosed MM (DT: mean 5.7), and treated MM (DT: mean 5.3), no significant differences were found (*p* = 0.26). In multivariate analysis, compared with the premalignant stages (reference), no statistically significant associations of distress were found between a new diagnosis of MM (*p* = 0.87) or treated MM (*p* = 0.33).

No significant differences were found for anxiety symptom scores between the three groups (MGUS or SMM: mean 4.1/newly diagnosed MM: mean 4.8/treated MM: mean 4.6; *p* = 0.21). The results of multivariate analysis confirmed these findings (Table 2). Compared with the premalignant stages (reference), no statistically significant associations of anxiety symptom scores were found between newly diagnosed MM (*p* = 0.6) or treated MM (*p* = 0.91). For depression symptom scores, significant differences were found between the groups (MGUS or SMM: mean 4.9/newly diagnosed MM: mean 5.6/treated MM: mean 6.9; *p* < 0.001, respectively). In multivariate regression analysis, there was no association between newly diagnosed MM and depression score (*p* = 0.98), although a significant association was found between treated MM (*B* = 1.3; *p* = 0.04) when compared with the precursor stages (MGUS/SMM; reference).

To the best of our knowledge, this is the first investigation of HRQOL and various aspects of mental health considering precursor states of MM in a large clinical sample of over 500 patients. In a recently published article with a smaller sample of 292 participants we investigated the relation between resilience, HRQOL and depression in MM and its premalignant stages. Patients with a high level of resilience had a better HRQOL. In this study DT and anxiety levels were not considered^11^. Patients’ experience in terms of quality of life need to be considered in a group of individuals who have a life expectancy that is not significantly limited by the respective diagnosis, as in the case of MGUS. In an ongoing longitudinal study, launched in November 2016 in Iceland, aiming to include ~120,000 adults for a screening to identify precursors of MM, one aim is to determine the optimal diagnostic procedures for individuals with MGUS. Longitudinal investigations of HRQOL are to be included as part of this study. However, the results will not be available in the near future^12^.

In our study, patients with MM reported lower levels of physical HRQOL compared with patients with a precursor disease. Lowered levels of PCS in patients with MM compared with precursors in our sample can be explained by the typical symptoms of active MM, including fatigue and pain, or by treatment-related side effects^13^.

In our study, no significant differences in mental HRQOL, distress, or severity of anxiety symptoms were found between the precursors and MM groups in univariate or multivariate analyses. This is in line with the reports of clinicians that the psychological burden of cancer anticipation and screening procedures in MGUS leads to similar psychological burdens for patients with MGUS, SMM, and MM alike^6^.

In our sample, ~63% of all participants reported levels of DT above the cutoff-score of ≥5 with no significant differences between subgroups, suggesting a need for psychosocial support in the majority of patients.

The prevalence of clinically relevant anxiety symptoms in MM was comparable to other studies in the field^14^. The fact that there was no significant differences between precursors and active MM suggests the need to screen for psychological burden in this vulnerable group.

Levels of depression symptoms were significantly higher in the group with treated myeloma than in the precursor group. This may be due to treatment-related adverse events and disease-related symptoms.

The major strength of the present study is the large clinical sample. HRQOL, DT, anxiety, and depression were measured with validated questionnaires. The impact of our findings is magnified by the fact that the clinical prevalence (diagnosed cases) of MGUS is huge—estimated to be >500,000 in the US alone^15^.

The main limitation is related to its cross-sectional design, which does not allow for either temporal or causal inferences.

Our findings support the perception of many clinicians who report patients with MGUS and SMM suffering significantly from the knowledge of their risk of developing MM. Therefore, even in patients with a precursor disease, it may be important to routinely measure DT in order to provide needs-based support.

## Supplementary information

Results of univariate analyses

STROBE Flow Diagram
