# Supplementary material for: Patients with precursor disease exhibit similar psychological distress and mental HRQOL as patients with active myeloma
Source: Blood Cancer J. 2019 Jan 21;9(2):9. doi: 10.1038/s41408-019-0172-1 (PMC6341083; doi:10.1038/s41408-019-0172-1)
Supplement: Supplementary file 1 — Results of univariate analyses [file 41408_2019_172_MOESM1_ESM.docx]

**Supplementary Table: Group comparisons (Groups 1-3) of means of health-related quality of life, psychological distress, anxiety, and depression assessed using Kruskal–Wallis tests.**

| Variable | Precursor disease (MGUS. SMM) M (SD) | New diagnosis M (SD) | Treated MM M (SD) | *P* |
| --- | --- | --- | --- | --- |
| Health-related quality of life |  |  |  |  |
| Physical Component Score | 43.6 (10.4) | 39.1 (11.3) | 35.7 (9.9) | <0.001 |
| Mental Component Score | 43.1 (11.3) | 42.9 (12.3) | 41.3 (11.1) | 0.18 |
| Distress | 5.2 (3.0) | 5.7 (2.8) | 5.3 (2.6) | 0.26 |
| Anxiety Score (GAD-7) | 4.1 (4.2) | 4.8 (4.8) | 4.6 (4.0) | 0.21 |
| Depression Score (PHQ-9) | 4.9 (4.6) | 5.6 (4.8) | 6.9 (4.2) | <0.001 |

*Legend:* MM, multiple myeloma; MGUS, monoclonal gammopathy of undetermined significance; SMM, smoldering myeloma; PCS, physical component score; MCS, mental component score; GAD-7, Generalized Anxiety Disorder Scale-7; PHQ-9, Patient Health Questionnaire-9.
