# Supplementary figures and images for: Patients with precursor disease exhibit similar psychological distress and mental HRQOL as patients with active myeloma
Source: Blood Cancer J. 2019 Jan 21;9(2):9. doi: 10.1038/s41408-019-0172-1 (PMC6341083; doi:10.1038/s41408-019-0172-1)

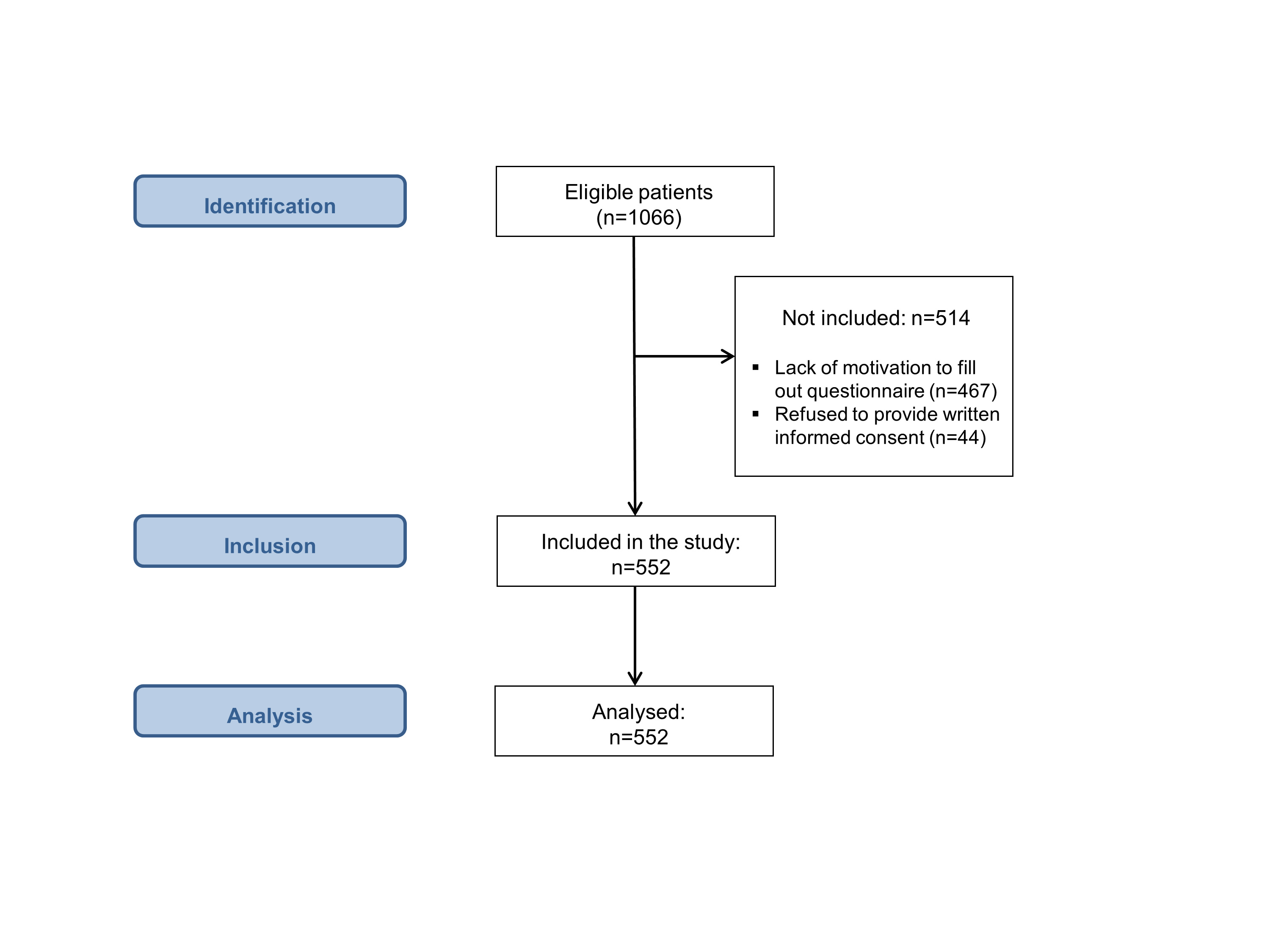

Supplement: Supplementary file 2 — STROBE Flow Diagram [file 41408_2019_172_MOESM2_ESM.tif]
